# A comparison of CPAP and CPAP_FLEX_ in the treatment of obstructive sleep apnea in World Trade Center responders: study protocol for a randomized controlled trial

**DOI:** 10.1186/s13063-015-0907-7

**Published:** 2015-09-10

**Authors:** Indu Ayappa, Jag Sunderram, Kathleen Black, Akosua Twumasi, Iris Udasin, Denise Harrison, Jeffrey L. Carson, Shou-En Lu, David M. Rapoport

**Affiliations:** Division of Pulmonary, Critical Care and Sleep Medicine, Department of Medicine, New York University School of Medicine, New York, NY USA; Department of Medicine, Rutgers Robert Wood Johnson Medical School, New Brunswick, NJ 08903 USA; Environmental and Occupational Health Sciences Institute, Rutgers Robert Wood Johnson Medical School, Piscataway, NJ USA; School of Public Health, Rutgers Robert Wood Johnson Medical School, Piscataway, NJ USA

**Keywords:** Obstructive sleep apnea, World Trade Center, Nasal resistance, Continuous positive airway pressure, Cflex, CPAP_Flex_

## Abstract

**Background:**

Following the World Trade Center disaster, a large number of individuals involved in rescue and recovery activity were exposed to significant amounts of dust, and reported symptoms of chronic nasal and sinus inflammation. An unusually high prevalence of obstructive sleep apnea (OSA) has also been observed in this World Trade Center Responder population. This project aims to examine the relationship between nasal pathology and OSA. Our hypothesis is that increased nasal resistance due to nasal inflammation predisposes to OSA in this population. Continuous Positive Airway Pressure (CPAP) is the standard therapy for OSA but despite its efficacy has poor adherence. Subjects with high nasal resistance may have greater difficulty in tolerating this therapy than those who do not have high nasal resistance. Reduction of excess expiratory positive pressure by the modality known as Cflex™ during Continuous Positive Airway Pressure therapy (CPAP_Flex_) has been suggested to improve comfort without compromising efficacy. We will compare CPAP to CPAP_Flex_ in subjects with OSA.

**Study Design:**

Subjects with new onset habitual snoring will be screened for OSA using home sleep studies and rhinomanometry will be used to determine nasal resistance. In 400 subjects with OSA we will perform a randomized double blind cross-over study comparing CPAP to CPAP_flex_, and relate nasal resistance to adherence to CPAP therapy.

**Discussion:**

This is the first multicenter trial designed to test the hypothesis that adherence to CPAP therapy relates to nasal resistance and CPAP_Flex_ will improve adherence to CPAP in those subjects with high nasal resistance.

We anticipate the following results from this trial: 1. Increased nasal resistance is associated with decreased adherence to CPAP therapy. 2. Use of CPAP_Flex_ improves adherence with CPAP therapy in subjects with high nasal resistance, but not in those with low nasal resistance. 3. The benefit of CPAP_Flex_ on adherence is greatest when offered at CPAP therapy initiation rather than as a “rescue” therapy in subjects with high nasal resistance.

**Trial Registration:**

ClinicalTrials.gov Identifier: NCT01753999, Date: 12 December 2012

## Background

Following the World Trade Center (WTC) disaster on 11 September 2001, an estimated 40,000 individuals including fire fighters, police and other public sector workers were exposed to significant amounts of dust while working in rescue, recovery and debris removal [1]. A medical screening program was developed to evaluate the health status of workers and volunteers who spent time at the WTC site and sustained exposure in the aftermath of 11 September.

The WTC Worker and Volunteer Medical Screening Program (MSP) and the follow-up World Trade Center Medical Monitoring and Treatment program (WTCMMP) now called the World trade Center Health program (WTCHP) have successfully recruited more than 27,000 responders to assess and treat health effects from these exposures [2]. About 1700 of these responders are followed at the Environmental and Occupational Health Sciences Institute (EOHSI) of Rutgers Robert Wood Johnson Medical School (RWJMS) in Piscataway, New Jersey and about 2100 in the NYU School of Medicine Clinical Center of Excellence (NYUSOM CCE) at Bellevue Hospital in New York City. Standardized questionnaires were completed by these responders. These questionnaires provide the medical history and presence of new symptoms following the exposure and the presence of diseases such as OSA.

The collapse of the WTC towers resulted in a massive plume of building debris and particulate dust that was highly alkaline and corrosive and contained high levels of calcium sulfate (gypsum) and calcium carbonate (calcite) that are capable of causing chemical irritation to the upper respiratory tract and irritation of mucous membranes [3, 4].

### Obstructive sleep apnea (OSA)

OSA is a chronic condition with recurrent episodes of partial or complete upper airway collapse during sleep. The main risk factors for OSA are obesity and male gender and it is highly prevalent in the general population, with estimates ranging from 5–10 % to > 25 % [5, 6]. Upper airway inflammation resulting in mucosal congestion and edema could increase nasal and posterior pharyngeal resistance and provide an alternate mechanism for development of compromised upper airway patency during sleep. We hypothesize that WTC dust exposure results in upper airway and nasal inflammation, leads to nasal symptoms and increased nasal resistance and ultimately to OSA.

The health benefits of diagnosis and treatment of OSA are well recognized: untreated OSA is associated with daytime sleepiness, increase in motor vehicle accidents, increased hypertension, stroke, impaired glucose metabolism, and increase in all-cause mortality [7, 8]. CPAP is the primary treatment for OSA [9]. CPAP use normalizes sleep architecture, reduces daytime sleepiness, and reduces automobile accidents and lowers blood pressure slightly (2–3 mmHg) and may reduce cardiovascular events [10–14]. Despite its efficacy, 29–83 % of patients are non-adherent to CPAP [15] and no specific factors (demographic, disease specific) that predict CPAP adherence have been identified. In addition to problems with the mask and claustrophobia, pressure intolerance and “difficulty exhaling” are frequently cited by patients as limiting acceptance of CPAP therapy [15]. Nasal symptoms and side-effects are also common and may account for 30–50 % of cases of CPAP intolerance [16]. Thus, in addition to its role in causing OSA [8], elevated nasal resistance may impact on initial acceptance of CPAP [17, 18]. Small studies [16] have shown that nasal resistance was significantly higher in OSA patients who did not tolerate CPAP and reduction of nasal resistance by surgery [19] has been shown to increase CPAP use. CPAP adherence in WTC responders with chronic rhinosinusitis is unknown but in general has anecdotally been thought to be poor. High nasal resistance can play a potential role in their poor adherence. CPAP keeps the upper airway open and nasal resistance becomes the primary determinant of total upper airway resistance. Thus, during CPAP use, high nasal resistance may continue to cause a patient to experience discomfort while exhaling despite adequate relief of OSA and could contribute to intolerance of CPAP. In preliminary data we have shown that, as expected, increased nasal resistance results in higher expiratory pressure. By decreasing this excess pressure during the expiratory cycle, CPAP_Flex_ (Philips Respironics, Murry Ridge Lane, Murrysville, PA) may improve CPAP adherence. Although prospective, randomized studies have demonstrated that CPAP_Flex_ is not inferior to conventional fixed CPAP, increased adherence has not been uniformly demonstrated. Some studies have shown CPAP_Flex_ reduces discomfort and improves adherence [20], but larger randomized studies [21] have shown no difference in adherence between CPAP and CPAP_Flex_. However, none of these studies have attempted to target therapy to patients based on elevated nasal resistance as we propose to do.

### Study rationale

In this study we will relate nasal resistance to CPAP adherence in patients with OSA and show that reduction of expiratory pressure using CPAP_Flex_ will improve CPAP adherence. Patients with OSA will be randomized in a double blind cross-over design to receive CPAP or CPAP_Flex_ and adherence will be measured.

We will test the hypotheses that:

- Increased nasal resistance is associated with decreased adherence to CPAP.
- Use of CPAP_Flex_ will improve adherence with CPAP in subjects with high nasal resistance, but not in those with low nasal resistance.
- The benefit of CPAP_Flex_ on adherence will be greatest if it is offered at CPAP initiation rather than as a “rescue” therapy in subjects with high nasal resistance.

## Methods/Design

### Design and setting

**Fig. 1:**
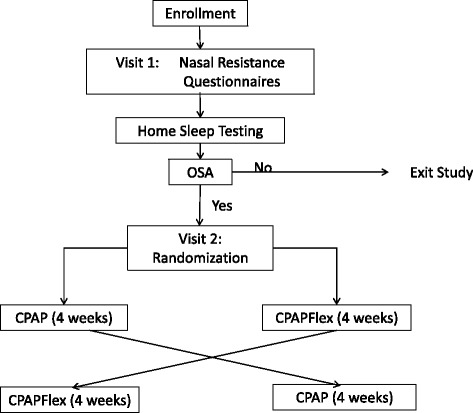
Protocol flow chart. Simplified illustration of the protocol for all participants. CPAP, continuous positive airway pressure; CPAPFlex, continuous positive airway pressure with reduced pressure during expiration OSA, obstructive sleep apnea

This study is a two-center double blind randomized control trial with a cross-over design in WTC responders with OSA comparing CPAP to CPAP_Flex_ (See Fig. 1)

.

### Ethical aspects

The study protocol and patient information documents have been approved by the Institutional Review Board of both NYU School of Medicine (NYU SOM, IRB Protocol #S12-02578) and Rutgers Robert Wood Johnson Medical School (Rutgers RWJMS, IRB Protocol #2012002164) and the Population Protection Committee of the WTCHP. Informed consent is obtained from each subject prior to participation in the study.

#### Study population and clinical evaluation

We will study a total of 400 subjects with OSA (200 at each site) from the Clinical Centers of Excellence (CCE) for responders (*n* = 2100) at NYU SOM and at EOHSI, Rutgers RWJMS (*n* = 1700). Recruitment for the study began in March of 2013 and will continue until August of 2016.

Inclusion criteria: 1) Member of the WTCHP at NYU SOM or Rutgers RWJMS, 2) OSA diagnosed on the basis of a 2-night home sleep study.

Exclusion criteria: (i) Gross skeletal alterations affecting the upper airway (eg. Micrognathia), (ii) Unstable chronic medical conditions known to affect OSA (congestive heart failure, stroke), (iii) Pregnancy or intent to become pregnant within the period of the protocol, (iv) Inability to sign informed consent form, (v) habitual snorer or diagnosis of OSA prior to 11 September 2001.

### Objective assessment of nasal pathology

#### Clinical examination

Anterior rhinoscopy is performed to identify erythema, edema, polyps or polypoid swelling, crusting, mucin, and or frank pus and documented.

#### Rhinomanometry

In 2005, the Standardization Committee on Objective Assessment of the Nasal Airway recommended that 4-phase rhinomanometry be adopted as the universal standard for measurement of nasal resistance [22]. It consists of simultaneous measurement of airflow through the nose and differential pressure required for its generation. By dividing inspiration and expiration into increasing and decreasing phase, high-resolution rhinomanometry (HRR) introduces two new parameters: effective resistance and vertex resistance and has been validated in normal and pathological individuals. We assess nasal resistance using the 4-phase-rhinomanometer (RhinoLab GmbH, Rendsburg, Germany) following the detailed technical, practical methodology, and referenced normative data published in the literature [22]. Measurements are performed seated and in the supine position (before and 10 minutes after decongestion with 0.1 % xylometazoline solution) in a quiet procedure room with a constant temperature. Parameters obtained from the HRR include the unilateral effective and vertex resistances during inspiration, expiration and during the entire breath. “Effective Resistance (Reff)” describes the computerized measurement and calculation of 2000 effective flow and differential pressure measurements recorded for each averaged breath. “Vertex Resistance” is the resistance of the nasal airstream at the point of maximum flow during inspiration or expiration in a breath. Total nasal resistance is calculated using Ohm’s law modeling the nose as parallel resistors using the following formula:

$$ \mathrm{Total}\ \mathrm{Nasal}\ \mathrm{Resistance} = \mathrm{Rright}\ *\ \mathrm{Rleft}/\left(\mathrm{Rright} + \mathrm{Rleft}\right). $$

Effective and vertex resistance will be presented after the recommended logarithmic transformation. Log Reff ≤ 0.8 is defined as low resistance and > 0.8 as moderate-high resistance.

#### Home monitoring for OSA

Subjects are given an ARES™ Unicorder (Watermark Medical Inc., West Palm Beach, FL, USA) to take home and wear for two nights, with a pre-addressed mailer to return the device to the sleep lab. The ARES Unicorder is worn on the forehead and does not require additional wires to external devices. It measures oxygen saturation and pulse rate from reflectance oximetry, airflow from a nasal cannula/pressure transducer, snoring via acoustic microphone and head movement actigraphy and head position from accelerometers. The device also provides audible alerts during the study if poor quality airflow or SpO_2_ is detected so the subject can reposition the device.

#### Analysis of respiratory data from ARES

Data from the monitor is autoscored and then manually reviewed by a single trained sleep technician at NYU SOM. There has been significant evolution of the definition of OSA in the epidemiological literature since the landmark study of Young et al. [5], where a reported incidence of 2–4 % was based on an Apnea + Hypopnea Index (AHI) > 5/hour and symptoms of excessive daytime somnolence. Apneas are scored when there is a reduction in airflow to less than 10 % of baseline. Hypopneas4 % are scored for > 30 % reduction in airflow associated with 4 % or more decrease in oxygen saturation. In most of the studies since, including the Sleep Heart Health Study [23], hypopnea has been defined as a reduction in airflow amplitude associated with desaturation and/or arousal, but the prevalence of OSA is five-fold greater when arousals are included without associated desaturation. The American Academy of Sleep Medicine has issued several definitions of OSA, most recently in 2007 [24] but all require an electroencephalogram (EEG) and there is no published consensus as to how to score home monitoring without EEG. The approach we take is to use AHI4 % (nearly identical to the recommended AHI of the AASM AHI) and to define the Respiratory Disturbance Index (RDI) as the sum of AHI4 % and the more subtle respiratory events (Hypopnea 1 %, which approximates the AASM “RERAs” or Respiratory Effort Related Arousals defined with an EEG). Hypopneas 1 % are scored for > 30 % reduction in airflow associated with 1 % decrease in oxygen saturation/or surrogate of arousal. AHI 4 % is calculated as apneas + hypopneas4 % divided by total valid recording time. The RDI is calculated as apneas + hypopneas4 % + hypopneas1 % divided by total valid recording time. Using these metrics, we define OSA as present when AHI 4 % > 5/hour or when RDI > 15/hour. We have extensive experience using the ARES device and have validated it against NPSG in over 300 subjects in multiple research studies [25, 26]. In our published validation study the diagnostic sensitivity for diagnosing OSA using a cutoff for RDI of 15/hour ranged from 85–95 % and specificity from 91–94 %. Of particular note the failure rate for acquiring scorable data is < 10 % [25].

We have previously compared ambulatory studies performed with the ARES to laboratory NPSG [25, 27], and shown that, with the ARES, AHI4% is well measured and AHI1% is functionally equivalent to the RDI.

### Intervention

1. From the ambulatory ARES studies, we expect to identify approximately 750 subjects with OSA and plan on recruiting 400 subjects for the CPAP/CPAP_Flex_ trial. All subjects willing to try CPAP are recruited to undergo titration and measures of nasal resistance. Participants are randomly assigned to CPAP or CPAP_Flex_ as a first treatment, and crossed over to the other after completing the first arm of the protocol.
2. Titration of the assigned therapy is in the home with AutoCPAP with CPAP or CPAP_Flex_ over 5 days. Prescribed therapy is at the 90th centile of autotitration pressure after this period if the subject uses the machine for more than 4 hours a night on each of those nights. If fixed pressures are not achieved by the end of the first week, the subject is left in the autotitration mode.
3. Adherence is continuously monitored via the modem connected to the CPAP and/or the CPAP compliance card for 1 month with first intervention, then switched to the alternate intervention and adherence monitored for another month. Data from the last 2 weeks of each period will be used for objective adherence comparisons between interventions.
4. Subjective assessment of sleepiness, quality of life and satisfaction with therapy are obtained using questionnaires administered at the start and end of each period.

#### Initiation of therapy

After results of the ambulatory monitoring identify OSA, subjects return to the study center for discussion with study staff regarding therapy. If they agree to CPAP, they undergo mask-fitting and desensitization and CPAP education during this visit. CPAP education follows a written protocol common to both sites. During the first week, the research co-coordinator calls the patient twice (day 3 and day 7) to discuss any mask or CPAP-related issues. Period 1 of the intervention begins subsequent to this break-in week and lasts for 4 weeks. Treatment is switched remotely and period 2 of the intervention begins subsequent to that and last 4 weeks.

#### Randomization and blinding

OSA patients are randomly allocated to CPAP or CPAP_Flex_stratified by site and low (log[Reff] ≤ 0.8) and high (log[Reff] > 0.8) resistance, gender and AHI (> or < 30 events per hour of sleep) by the statistician. This is accomplished by a computer generated allocation table of randomly permuted blocks of assignments to study condition (CPAP or CPAP_Flex_) for each site. Outcome of the randomized choice of treatment is provided to an unblinded individual who is not part of the analysis of the study data, when a subject arrives to the center, and who remotely sets the allocated CPAP device at the start of each treatment period. Devices are tracked by serial number. Trial participants are blinded to the therapy type but may be able to perceive a difference in the mask pressure during expiration. CPAP and CPAP_Flex_ machines are identical otherwise and no feedback is provided to subjects regarding the type of treatment. All investigators and research personnel who enroll and interact with the participants, outcomes assessors and data analysts are blinded to the intervention and allocation as it is remotely set and not evident on the device. The unblinded individual (not the PI) maintains a master password protected file containing the serial number, patient ID and designation code for each treatment period and subject. Except in a medical emergency situation this file will not be opened until the study has been completed and all data have been entered/cleaned. The unblinded individual will document any premature unblinding that may occur. The unblinded individual also reviews the data relevant to treatment efficacy during the first 5 days of autotitration and significant deviations from prescribed pressure due to leak during the rest of the intervention period.

#### AutoCPAP titration

All subjects diagnosed with OSA are given an autotitrating machine. We use the Respironics AutoCPAP device which has capability of autotitration of pressure with or without Cflex set at a level 3. The devices communicate with the sleep centers via modem or broadband (HIPPAA- compliant). The auto-titrating positive airway pressure (APAP) with a range of pressures from 5–15 cm H_2_O *with or without* CPAPflex is set when the subject presses a modem button after turning on the machine at home. These machines have been used extensively [28, 29] and are used to determine a single optimal pressure after a period of autotitration. During the first 5 days of autotitration, summary raw airflow data collected are reviewed by an unblinded technician (see blinding above) to ensure efficacy of treatment. After 5 nights of at least 4 hours of use, the device automatically switches to a fixed pressure from the 90th percentile of pressures achieved and used for the remainder of the study. Patients who are non-compliant (use for less than 4 hours a night) during the first 5 nights remain on autotitrating pressures. The automated AHI is monitored to ensure efficacy of therapy (AHI of < 10 events per hour of sleep). If the AHI is > 10 events per hour of sleep the data is reviewed by a designated unblinded sleep physician and the pressure is modified or the patient is recommended for an in laboratory sleep study if indicated. All patients are provided heated humidification.

### Primary outcome measure

In this intention-to-treat study, primary data to be collected will be adherence with therapy (hours of usage at set pressure) per night, and residual AHI on treatment. Those patients who are diagnosed with OSA but who refuse to come in for CPAP therapy will be excluded from analysis. Primary outcome measure will be mean hours of usage at set pressure per night during the last 2 weeks of the intervention.

#### Monitoring of CPAP efficacy and adherence

Efficacy will be evaluated by (i) reviewing residual AHI and inspiratory flow limitation at optimal pressure as recorded on the device and (ii) review of raw airflow signal. Nightly adherence at the optimal pressure is recorded on the device and also transmitted to the sleep center onto a HIPPAA-compliant website that is password protected.

#### Cross-over to alternate therapy

Four weeks after the fixed pressure is achieved following the first intervention (ie CPAP or CPAP_Flex_), the device will be switched to the alternate mode by remote modem connection and the autotrial is started again. This duration was chosen because long-term CPAP adherence has been consistently shown to be predicted primarily by early short-term CPAP adherence [30–33].

#### Subjective assessment

At the end of each intervention period subjects will fill out the Epworth Sleepiness Scale (ESS), Functional Outcome of Sleep Questionnaire (FOSQ) and a satisfaction questionnaire [29].

### Analysis plan and statistical considerations

#### Power analysis

We will randomize 400 subjects with OSA, stratified by nasal resistance, into two sequences of treatment: CPAP_Flex_ followed by CPAP alone versus CPAP alone followed by CPAP_Flex_. To determine if high nasal resistance is associated with decreased CPAP adherence, we based our power analysis on the method of Fisher’s Z test [34] for correlation coefficients. With 400 participants and setting alpha at 0.05 (2-sided), we will have 80 % power to test a small (negative) correlation of −0.14. To determine if use of CPAP_Flex_ will improve adherence with CPAP in subjects with high nasal resistance, but not in those with low nasal resistance, we assume that 50 % of our subjects (200) will have high nasal resistance, based on our pilot data in non-WTC subjects. In Aloia et al. [20], they found that CPAP_Flex_ significantly improved adherence of CPAP at 4 weeks (3.5 ± 2.8 hours for CPAP and 4.7 ± 2.2 hours for CPAP_Flex_ corresponding to Cohen’s *d* of 0.49). Assume that the effect size of CPAP_Flex_ improvement between high versus low nasal resistance is 80 % of the effect of Aloia et al., Cohen’s *d* = 0.40, representing an improvement of 20 minutes per night assuming the SD of the improvement to be 60 minutes (in paired/cross-over studies SD is usually smaller than that in unpaired studies). With set power = 80 % and alpha = 5 % (2-sided), to test an effect size *d* = 0.40 we will need 100 subjects each with high and low nasal resistance, based on the method of a 2-sample *t* test with equal variance. With a total of 400 subjects, we have enough power to test this hypothesis.

To determine if the benefit of CPAP_Flex_ on adherence will be greatest if it is offered at CPAP initiation rather than as a “rescue” therapy in subjects with high nasal resistance, we expect that 200 subjects will have high nasal resistance and half (100) of them will receive CPAP_Flex_ at the initiation of treatment per randomization. Using the method of a 2-sample *t* test, we will have 80 % power (alpha = 0.05, 2-sided) to test a difference of 23.9 minutes (SD = 60 minutes) in improvement between subjects with high nasal resistance and CPAP_Flex_ versus CPAP at initiation. After accounting for 40 % CPAP rejection, we will be able to test a difference of 27.6 minutes (SD = 60 minutes) in the CPAP_Flex_ improvement.

#### Statistical analysis plan

To determine if high nasal resistance is associated with decreased CPAP adherence we will first explore the distributions of nasal resistance and adherence of CPAP (number of hours/night), both in continuous scale and the discrete scale (nasal resistance (high versus low at the cutoff of log[Reff] = 0.8); CPAP rejection (rejection “yes” versus “no” at a cutoff of 2 hours/night). We will calculate the correlation between nasal resistance and CPAP when both are treated as continuous measures, and use the chi-square test when both are discrete variables. To control for age, gender, BMI, and AHI we will use the linear regression (in continuous case) and logistic regression (in discrete case) analyses as we evaluate these associations. To determine if the use of CPAP_Flex_ will improve adherence with CPAP in subjects with high nasal resistance, but not in those with low nasal resistance, we will use mixed model analysis for repeated measures resulting from the cross-over design. Specifically, we will use the average hours of CPAP/CPAP_Flex_ use over the last 2 weeks in each phase as the dependent variable. Treatment assignment (CPAP versus CPAP_Flex)_, nasal resistance (high versus low according to the cutoff of log[Reff] = 0.8) and their interaction will be modeled as fixed effects in the statistical model and the intra-subject correlation between repeated measures will be modeled using random effects. If the use of CPAP_Flex_ improves adherence to CPAP in subjects with high nasal resistance, but not in those with low nasal resistance, we expect to observe a significant interaction of treatment assignment and nasal resistance. Linear contrasts will also be constructed to evaluate improvement with CPAP_Flex_ in subjects with high and low nasal resistance separately. Variables such as age, gender, BMI, AHI, etc., will be further controlled for in these statistical analyses. Finally, to determine if the benefit of CPAP_Flex_ on adherence will be greatest if it is offered at CPAP initiation rather than as a “rescue” therapy in subjects with high nasal resistance, we will use linear regression analysis with the difference in the average use between CPAP and CPAP_Flex_ as the dependent variable, treatment sequence (CPAP followed by CPAP_Flex_ versus CPAP_Flex_ followed by CPAP), nasal resistance (high versus low) and their interaction as the independent variables. If the benefit of CPAP_Flex_ is greatest when it is offered at initiation in subjects with high nasal resistance, we expect the interaction of treatment sequence and nasal resistance to be significant. Linear contrasts will also be constructed to evaluate improvement with CPAP_Flex_ for each combination of treatment sequence and nasal resistance separately. If a substantial proportion of subjects reject CPAP and/or CPAP_Flex_ and results in excessive zeros (or low numbers close to zero) in the data, we will use the two-part model [35, 36] to analyze the data. In general, the term two-part model refers to having both a logistic model part to model the probability of non-zero versus zero outcomes (part 1) and a linear model part for the values of the non-zero outcomes (part 2). In our study, we will use the logistic model part to model the probability of rejection (defined by total CPAP/CPAP_Flex_time < 2 hours, “yes”/”no”), and the linear model part to model the averaged daily use in hours after excluding those who reject. Treatment assignment (CPAP versus CPAP_Flex_), nasal resistance (high versus low) and their interaction will be the independent variables in both model parts. Each hypothesis will be tested using either the score or the Wald test [36]. Subgroup analyses will be performed based on (i) sleep apnea severity (mild sleep apnea – AHI4% 5 to ≤ 15/hour and moderate/severe sleep apnea – AHI4% > 15/hour), (ii) baseline sleepiness (ESS < 10 and ≥ 10), (iii) excluding subjects who are using sedative drugs.

## Discussion

The present study is one arm of a larger study of WTC responders where the relationship of nasal pathology to OSA is being examined. We hope to recruit 1000 subjects for the larger study, 400 of whom with OSA will participate in the RCT of CPAP versus CPAP_Flex_. The RCT portion of the study should have broad impact on the management of OSA potentially caused by upper airway pathology resulting from toxic inhalation of WTC dust. We are using a validated portable monitor to evaluate large numbers of subjects specifically for the presence of OSA. We are addressing the important specific question of whether nasal pathology negatively impacts on the ability to use CPAP, with the potential that addressing this with modified CPAP will improve the therapeutic approach to OSA. The study is being performed in collaboration with the NYUSOM CCE for Responders (principal investigator (PI) Denise Harrison, MD) which follows 2100 subjects and EOHSI, Rutgers RWJMS, Piscataway (PI Iris Udasin, MD) which follows 1700 responders. The study will generate new knowledge about conditions common in WTC exposed individuals, including nasal pathology and OSA. By using a less costly but equally effective evaluation of sleep disordered breathing (limited channel portable monitoring) and automated CPAP initiation [37–39] it will improve access to care for individuals exposed to WTC dust. The objective measures of nasal resistance measurements will help answer whether the OSA is attributable to nasal consequences of WTC exposure. Adherence and efficacy of different therapies will be evaluated providing guidelines to physicians caring for these patients.

## Trial status

The trial is currently ongoing and subjects are being actively recruited for the study.

## Abbreviations

AHI: Apnea Hypopnea Index
AHI4 %: Apnea Hypopnea4 % Index
AHI1 %: Apnea Hypopnea1 % Index
APAP: autotitrating positive airway pressure
BMI: body mass index
CCE: Center of Clinical Excellence
CPAP: continuous positive airway pressure
EEG: electroencephalogram
EOHSI: Environmental and Occupational Health Sciences Institute
ESS: Epworth Sleepiness Scale
FOSQ: Functional Outcome of Sleep Questionnaire
HRR: high resolution rhinomanometry
NYUSOM: New York University School of Medicine
PI: principal investigator
RDI: Respiratory Disturbance Index
Reff: Effective Resistance
RERA: Respiratory Effort Related Arousals
RWJMS: Robert Wood Johnson Medical School
SpO_2_: Oxygen Saturation
WTC: World Trade Center
WTCHP: World Trade Center Health Program
WTCMMTP: World Trade Center Medical Monitoring and Treatment Program
